# Back to the future of organolanthanide chemistry†

**DOI:** 10.1039/d2sc05976b

**Published:** 2022-11-30

**Authors:** Nolwenn Mahieu, Jakub Piątkowski, Thomas Simler, Grégory Nocton

**Affiliations:** a LCM, CNRS, Ecole Polytechnique, Institut Polytechnique de Paris, Route de Saclay 91120 Palaiseau France gregory.nocton@polytechnique.edu thomas.simler@polytechnique.edu

## Abstract

At the dawn of the development of structural organometallic chemistry, soon after the discovery of ferrocene, the description of the LnCp_3_ complexes, featuring large and mostly trivalent lanthanide ions, was rather original and sparked curiosity. Yet, the interest in these new architectures rapidly dwindled due to the electrostatic nature of the bonding between π-aromatic ligands and 4f-elements. Almost 70 years later, it is interesting to focus on how the discipline has evolved in various directions with the reports of multiple catalytic reactivities, remarkable potential in small molecule activation, and the development of rich redox chemistry. Aside from chemical reactivity, a better understanding of their singular electronic nature – not precisely as simplistic as anticipated – has been crucial for developing tailored compounds with adapted magnetic anisotropy or high fluorescence properties that have witnessed significant popularity in recent years. Future developments shall greatly benefit from the detailed reactivity, structural and physical chemistry studies, particularly in photochemistry, electro- or photoelectrocatalysis of inert small molecules, and manipulating the spins' coherence in quantum technology.

## Abstract 2

By taking inspiration from the structures and reactivities of its past, organolanthanide chemistry has managed to reinvent itself for the challenges of today and the future.

## Graphical abstract

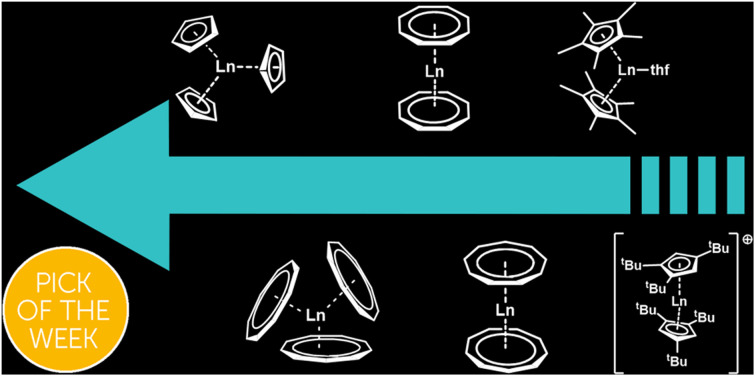

## Introduction

**Fig. 1:**
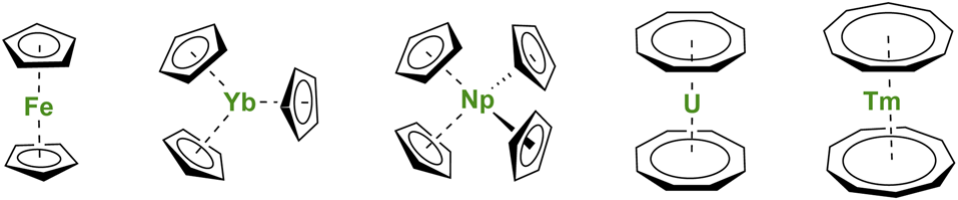
Molecular structures of ferrocene, Yb(Cp)_3_, Np(Cp)_4_, uranocene, and thulocene with unsubstituted C_*n*_H_*n*_ aromatic ligands.

During the winter of 1951, reading the journal *Nature* surprised many chemists.^1^ A new type of iron compound had been discovered (Fig. 1), and, with this discovery, a fantastic scientific adventure began. This story would culminate (but not end) with a Nobel Prize in 1973 for organometallic sandwich compounds. As several witnesses remember, the new structure at that time brought many questions related to the effects of π-coordination on the symmetry and physical properties of the complexes.^2–4^ A new field, similar to coordination chemistry, opened but with carbon-based ligands. The genesis of the metallocene success story lies in the chemical properties of the cyclopentadienyl (Cp) ligand, a small cyclic and mono-anionic ligand featuring 6π-electron Hückel aromaticity. Besides, this ligand framework is easy to modify and relatively robust.

Soon after the first report of unusual sandwich structures with the Cp ligand, several research groups became motivated to investigate its coordination behavior towards most metals of the periodic table, including, already at a very early stage, lanthanide ions.^5–7^ However, the result was not surprising, and several conclusions were drawn: (a) lanthanide ions are mostly trivalent with significantly larger ionic radii than most transition metal ions, and three Cp ligands easily fit around the metal center; (b) the metal–ligand bonding is principally ionic as demonstrated by the easy Cp ligand exchange from Ln(Cp)_3_ to iron halide to form ferrocene.^5^ The known divalent lanthanide analogs, Ln(Cp)_2_,^8,9^ are forming polymeric assemblies in the solid state to accommodate empty coordination sites with ligand electronic density, but these weak interactions can be broken by solvation.^10–12^ In all cases, the Cp ligands remain bent due to the bonding and respective sizes of the ligand and metal ion, as well as attractive dispersion or van der Waals interactions.^13–15^ The lanthanide series was completed with few of the actinides with similar findings,^16^ although the tetravalent states of Np or U allowed the wrapping of up to four Cp ligands around the metal centers (Fig. 1).^17–20^ In the original articles by Wilkinson, the magnetic moments of several Ln(Cp)_3_ complexes were measured at room temperature, and no significant deviations from the expected values were reported, except for the ytterbium complex Yb(Cp)_3_ (Fig. 1) exhibiting an unexpectedly low room temperature moment.^6^ This particular feature has been later explained by Denning *et al.*^21^ and relates to an unusual intermediate valent electronic structure for these, in appearance only, simple molecules.^22^

The size mismatch between the small cyclopentadienyl ligand and the large metallic f-elements has been easily corrected by increasing the size of the aromatic ligand used, either by substituting the hydrogens with bulkier groups^23,24^ or using larger aromatic rings.^25,26^ Both methods proved very efficient in accessing sandwich compounds of various charges and oxidation states with f-elements.

First, the use of large ligands such as the C_8_H_8_ ring, the dianionic cyclooctatetraenyl ligand (Cot), with uranium led to the formation of uranocene, U(Cot)_2_, a linear sandwich with a +4 metal oxidation state (Fig. 1). In the report from 1968, Streitwieser stated that the ligand size was nicely adapted to the f-orbitals, a situation similar to that found in between iron and Cp ligands.^27^ Lanthanide complexes supported by Cot ligands have been synthesized, but because of their predominant +3 oxidation state, the complexes are not neutral but anionic.^28,29^ The only exception to date is cerocene, Ce(Cot)_2_, the only neutral +4 complex of this family.^30,31^ The oxidation state in cerocene is also considered as intermediate valent,^22,32,33^ yet the overall complex remains neutral. The size of the aromatic ligand can be further increased to the C_9_H_9_ ring, corresponding to the monoanionic cyclononatetraenyl ligand (Cnt), which can be used with divalent Sm, Eu, Tm (Fig. 1) and Yb to form linear neutral sandwiches of 4f elements. Although the synthesis of the Cnt ligand was already reported in 1963,^34,35^ the first lanthanidocene Ln(Cnt)_2_ complexes were published in 2017 and 2018,^36,37^*i.e.* 50 years after the first report of uranocene. Note that the flexibility of the Cnt ligand also allows the formation of Ln(Cnt)_3_ complexes (*vide infra*).^38^

The neutral 4f-element sandwich complexes with unsubstituted aromatic ligands often suffer from poor solubility in organic solvents, which hinders both their characterization and the study of their reactivity. As such, derivatives of the versatile Cp and Cot ligands were used and constitute most of the sandwich complexes made in this area.^23,25^

Once the novelty of those arrangements wore off, the structural properties were primarily designed for the reactivity of the complexes. One key example is the development of the pentamethylcyclopentadienyl ligand (Cp*) and the corresponding Sm^II^ and Yb^II^ complexes as base-free or solvate adduct versions. Their vibrant chemistry prompted a generation of chemists to navigate through their stoichiometric reductive reactivity and catalytic properties, including in polymerization reactions.

**Fig. 2:**
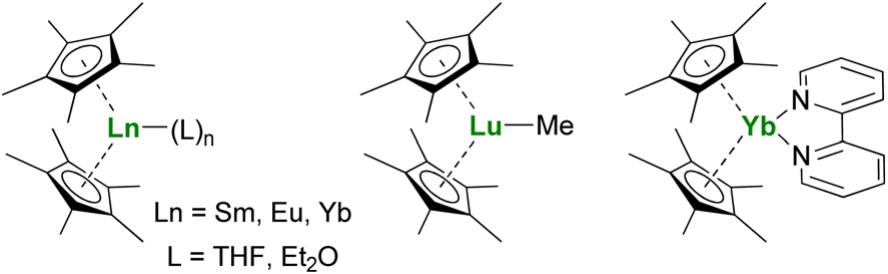
Molecular structures of Ln(Cp*)_2_ adducts, (Cp*)_2_LuMe, and (Cp*)_2_Yb(bipy).

The Ln(Cp*)_2_ complexes are bent sandwich compounds, which adducts of THF or diethyl ether were reported in the early 1980s (Fig. 2).^39,40^ The reduction potential of the Sm^II^ compound is lower than that of the Yb^II^ analog. It allows the reduction of CO,^41^ CO_2_,^42^ and even the non-polar and inert dinitrogen molecule: one N_2_ molecule can be reduced twice from two Sm(Cp*)_2_ fragments.^43^ The ytterbium complex is less reductive, yet it can easily reduce N-heteroaromatic fragments, such as bipyridine derivatives. The (Cp*)_2_Yb(bipy) complex was made originally in the early 1980s but only published in 2002.^44^ Indeed, during those years, its electronic structure remained difficult to rationalize until L_III_-edge XANES measurements and CASSCF computations pointed towards intermediate valent states.^45–48^ Other examples of N-heteroaromatic cycles followed,^48–52^ contributing to straightening the rationalization of the single electron transfer in ytterbium complexes.^22^

The reactivity of divalent organolanthanides extends to catalytic reactions, such as in ethylene polymerization,^53,54^ while the trivalent alkyl complex (Cp*)_2_LuMe (Fig. 2) reacts with methane,^55,56^ showing the vast scope of possible reactivity with one ligand set.

**Fig. 3:**
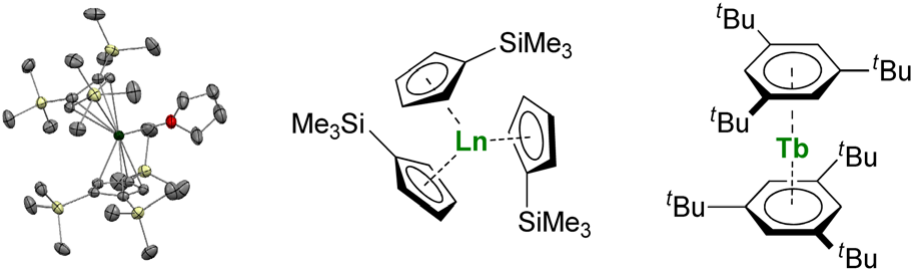
ORTEP plot of Tm(Cp′′′)_2_(thf) (Tm atom is in green, carbon in grey, silicon in yellow, and hydrogen are removed for clarity) and molecular structures of Ln(Cp′)_3_ and Tb(Bz^ttt^)_2_.

Larger substituents on the Cp ligands are also useful to stabilize kinetically “non-classical divalent lanthanides”^57^ as sandwich complexes of Tm (Fig. 3),^58,59^ Dy,^60^ and Nd,^61^ which still retain high reactivity for potential applications in the activation of small and inert molecules.^61,62^ Additionally, Lappert showed that using bulky substituents on the Cp rings from the original Ln(Cp)_3_ complexes allowed more straightforward reduction to form divalent lanthanide ions.^63,64^ This strategy has been more recently extended to almost all the lanthanide ions, even the most difficult to reduce, using multiple bulky Cp-derived ligands such as η^5^-C_5_H_4_(SiMe_3_) (Cp′) (Fig. 3), and showing the role of the ligand in the control of the redox properties of the lanthanide ion.^65,66^ Upon reduction, if the classical divalent lanthanide ions adopt a 4f^*n*+1^ configuration, in the other 4f-ions, the extra electron is promoted to the d-shell, opening a vast area of new applications for the redox chemistry of organolanthanides.

When the steric pressure induced by the ligands around the metal center reaches a certain level, the bulky ligands may enable an electron transfer to provide reductive chemistry, described as “sterically induced reduction”.^67–69^ A possible electronic contribution to these spontaneous reductions was also considered in the report of the Ln(Cnt)_3_ complexes. The corresponding synthesis was impossible for Sm or Yb but could be performed for the Tm and Y analogs.^38^ The original structures of tris-Cp arrangements made in 1954 found new horizons in the most recent studies.

Alongside reactivity studies, the fast development of molecules behaving as permanent magnets below a given temperature (Single Molecule Magnets, SMMs) became attractive.^70^ Rinehart and Long highlighted the relation between the ground m_*J*_ state of a given lanthanide metal ion and the coordination surroundings.^71^ Following this foundational perspective, the structural chemistry of 4f-organometallic sandwiches started to evolve for the formation of linear complexes with small and bulky Cp-based ligands,^72–75^ or large-size aromatic ligands,^76^ digging up the classic uranocene-like structures and building up the size of the Cp substituents' bulk from previous examples.^58,77,78^

Another great field evolution instance is the report of sandwich compounds supported by the neutral six-electron aromatic tris-*tert*-butylbenzene (Bz^ttt^) ligands. Using the metal vapor technique, Cloke was able to synthesize a few so-called zero valent neutral compounds (Fig. 3).^79–82^ Yet, the spectroscopic oxidation state and magnetic properties remain to be fully explored (*vide infra*) and may re-open the case of C_6_ rings with lanthanides.

The structural chemistry of organolanthanide complexes moved forward and back multiple times following the timely active area of applications, but the molecules resisted time. It is likely that a few abandoned molecules of the past will serve as figureheads for future developments. Synthetic innovation and rigorous methods thus remain the critical contribution. Along this perspective, we will attempt to focus on several possible horizons for organolanthanides.

## Reactivity and catalysis

### Light-promoted redox reactions

Photoinduced transformations, which encompass photoredox catalysis, are becoming increasingly popular methods to promote chemical transformations by taking advantage of light irradiation to alter the redox properties of compounds. This area of chemistry has long been governed by using photosensitizers based on rare and precious transition metals such as Ru and Ir.^83,84^ For sustainable chemistry, the development of earth-abundant and cheaper photocatalysts is a significant objective.^85^ Lanthanide-based photosensitizers can be considered promising candidates based on their unique optical properties and higher abundance in the Earth's crust compared to platinum group metals. It is worth noting that Ce, the most readily available lanthanide, features an abundance similar to that of the 3d metals Cu, Ni, and Zn. In contrast, the rarest lanthanides, Tm and Lu, are still more abundant than Ru and Ir.^86^

Upon light excitation, photosensitizers can behave as either potent reductants or oxidants, activating organic substrates through single electron transfer (SET) events, resulting in the formation of reactive radical intermediates. As a result of their partly filled 4f shell, most trivalent lanthanide ions absorb electromagnetic radiation in the visible region of the spectrum through parity-forbidden electronic transitions within the 4f shell. In addition to f–f transitions, electric-dipole allowed 4f^*n*^5d^0^ → 4f^*n*−1^5d^1^ transitions are also accessible but usually occur at much higher energies, typically in the UV region.^87^ The application of lanthanide complexes in light-promoted transformations, especially reduction reactions, strongly relies on such 4f → 5d transitions. Upon photoirradiation, the 5d excited state features a stronger reducing character, *i.e.* a more negative redox potential than the ground state, potentially allowing the reduction of challenging substrates typically not reduced under standard conditions without light irradiation.^88^ Although 4f orbitals are strongly shielded and remain largely unperturbed by the surrounding donor ligands, the 5d orbitals are sensitive to the ligand environment. Thus, the energies of the 4f → 5d transitions can be tuned depending on the nature and geometry of the surrounding ligands.^89^ In this context, organometallic ligands are exciting candidates as they usually enforce rigid and well-defined geometric coordination environments, potentially allowing tuning of the photophysical properties.

**Scheme 1:**
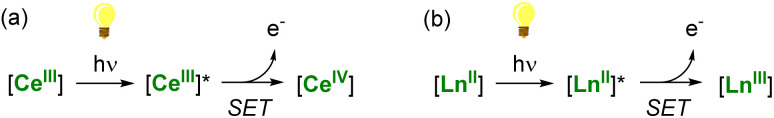
Light-promoted single-electron reduction reactions by trivalent (a) or divalent lanthanides (b).

A thermodynamically stable trivalent oxidation state characterizes all lanthanide ions. Only a few can easily shuttle between two oxidation states depending on their position in the lanthanide series and electronic configuration. The Ln^IV^/Ln^III^ couple is easily accessible for Ce, while the divalent oxidation state can be readily obtained in the case of Sm, Eu, and Yb complexes. These specific ions (Ce^III^, Sm^II^, Eu^II^, and Yb^II^) are excellent candidates for photoinduced reduction reactions and applications in (photo)catalysis through light-promoted amplification of their reducing properties (Scheme 1).

As a result of its 4f^1^ electronic configuration, the Ce^III^ ion gives rise to a broad 4f^1^ → 4f^0^5d^1^ transition in the near-UV/visible region and at the lowest energy compared to the other trivalent lanthanide ions. The energy of this transition involving 5d orbitals can be tuned by adjustment of the ligand environment.^90^ After photoexcitation, the redox-active Ce^3+^ ion acts as a highly reducing metalloradical that may participate in single-electron transfer (SET) reactions. The group of Schelter has primarily investigated the use of Ce^III^ complexes as photosensitizers for light-induced reductive transformations of organic compounds,^90^ including challenging substrates such as benzyl or aryl chlorides.^91–93^ The hexachlorocerate(iii) anion, [Ce^III^Cl_6_]^3−^, was especially found to be a potent photosensitizer with an estimated excited-state reduction potential of −3.45 V *vs.* Fc^+^/Fc (ferrocenium/ferrocene couple).^92^

To design efficient Ce^III^ photosensitizers, the relaxation of the excited state through nonradiative decay processes, such as ligand vibrational modes, should be minimized so that a maximum energy from light can be converted into chemical transformations.^94^ One strategy is to use rigid ligands and control C–H oscillators from proximity to the metal cation to minimize vibrational relaxation of the excited state. To this extent, developing new organocerium(iii) complexes may be interesting. The recent development of the Tb^IV^ and Pr^IV^ chemistry may also have an increasing interest in light of the Ce^IV^/Ce^III^ photochemistry.^95–100^ However, organometallic Tb or Pr complexes in the +4 oxidation state have not yet been reported.

**Scheme 2:**
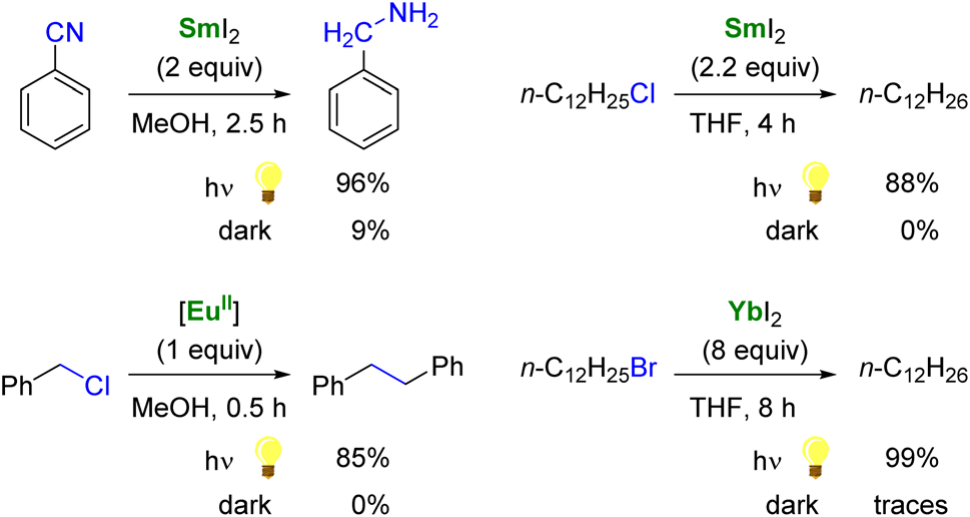
Examples of enhanced reductive reactivity of Ln^II^ (Ln = Sm, Eu, Yb) species upon photoirradiation.^108,110–113^

As single-electron reductants, divalent lanthanides, especially SmI_2_,^101,102^ have been extensively studied for their applications in organic chemistry.^103–105^ Compared to trivalent Ln ions, divalent lanthanides present 4f^*n*^5d^0^ → 4f^*n*−1^5d^1^ transitions at lower energies, typically in the UV to IR region.^106^ Photoexcitation to promote the 4f → 5d transition has been reported to enhance the rate of several reduction reactions mediated by divalent lanthanide species (Scheme 2).^88^ For example, the photoexcitation of SmI_2_ was found to lead to a more potent SET reductant, capable of reducing substrates such as organic chlorides and nitriles typically not affected by SmI_2_ without light irradiation.^107–110^ Similarly, although YbI_2_ is a weaker SET reagent with a redox potential *vs.* NHE of −1.15 V, photoirradiation in the near UV (300–400 nm) led to a more potent reductant with a reducing power similar to that of SmI_2_ in THF (−1.55 V).^111,112^ With a half-filled 4f^7^ ground-state electronic configuration, Eu^II^ ions are remarkably stable towards oxidation and show only a weak reducing character (−0.35 V *vs.* NHE). Recently, the group of Allen has demonstrated that a Eu^II^ complex supported by an azacryptand macrocyclic ligand (see below) could reduce organic chlorides upon photoexcitation in the visible region and exhibited an estimated reduction potential of −2.8 V *vs.* NHE, much more negative than that of SmI_2_.^113^

**Fig. 4:**
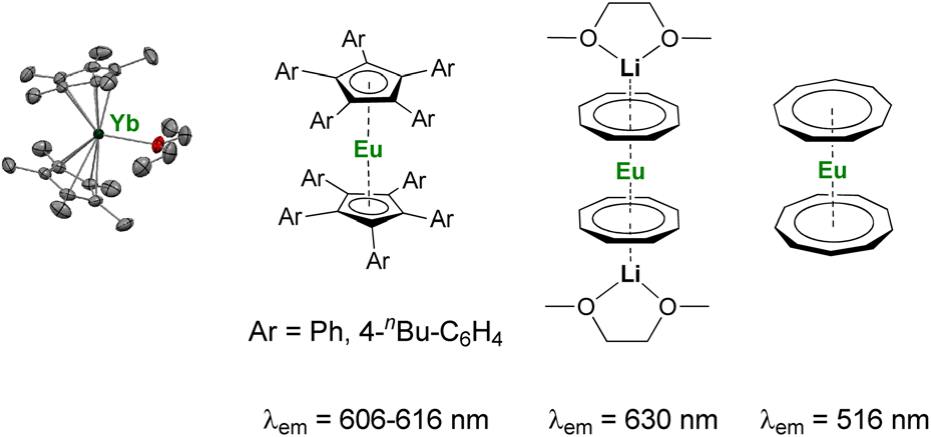
Example of organometallic divalent lanthanide complexes with potential applications in photoinduced reduction reactions.^36,40,116,117,119,120^

As discussed above, the organometallic chemistry of Yb^II^ and Sm^II^ complexes has been dominated by the Ln(Cp*)_2_(L)*_n_* (L = THF, Et_2_O; *n* = 0–2) complexes typically used for SET reactions. Although the group of Watson already reported in 1990 that the rate of C–F activation on fluorinated olefins and aromatics by Ln(Cp*)_2_(OEt_2_) (Ln = Yb, Eu) complexes could be enhanced upon visible-light irradiation,^114^ similar applications involving organolanthanide(ii) complexes have remained relatively unexplored. The interesting photophysical properties of Eu^II^ complexes^115^ have led to the recent synthesis and study of divalent organoeuropium complexes with tunable luminescence properties depending on the ligand environment (Fig. 4).^36,77,116–122^ With the recent introduction of novel organometallic architectures as supporting ligands, such as large ring ligands,^25,36,37^ further development in this field is likely to be expected. The development of ligand structures inducing long luminescence lifetimes is highly desirable for light-promoted photoredox reactions to maximize the probability of an electron transfer from the organolanthanide complex to the substrate.

For application in organic transformations, new organometallic reducing agents based on Eu^II^, Sm^II^, and Yb^II^ may offer alternatives to “non-classical divalent lanthanide” species, such as TmI_2_, DyI_2_ and NdI_2_, which feature stronger reducing properties but are more challenging to synthesize and handle.^57,123^ The primary focus in the organometallic chemistry of reductive divalent lanthanides is prone to go back to the early years (before 2000) when Eu^II^, Sm^II^, and Yb^II^ were the major representatives. Detailed studies of the ligand effects on the 4f → 5d transitions may lead to photoredox catalysts with greater tunability and negative electrochemical potentials. The remaining challenge in using divalent lanthanide complexes for photoredox catalysis is the reduction of the oxidized trivalent complexes back to their divalent states. A challenge is also the sensitivity toward air and moisture of organolanthanide complexes.

### Application in (photo)electrocatalysis

To use lanthanide complexes as SET reductants in catalytic transformations, different strategies can be used to regenerate the active, reducing species (Ce^III^, Sm^II^, Eu^II^, or Yb^II^).

**Scheme 3:**
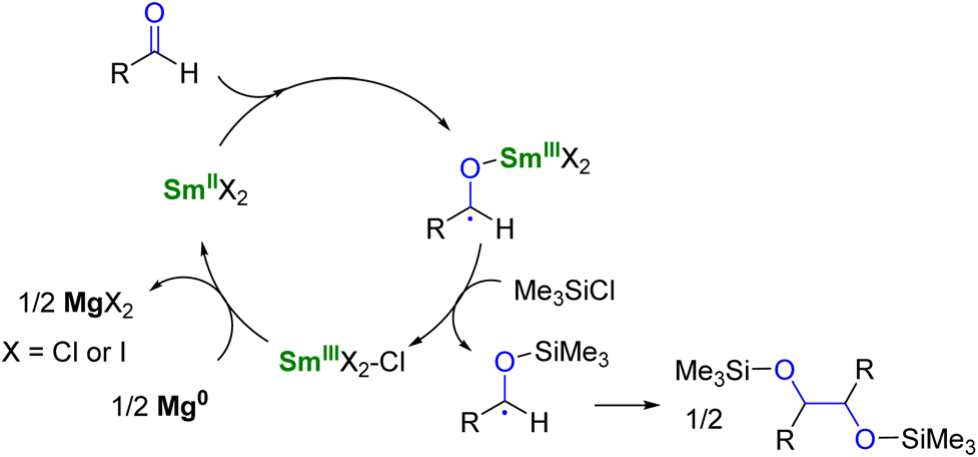
SmI_2_-catalyzed pinacol coupling reaction in the presence of Mg^0^ as co-reductant.^125^

**Scheme 4:**
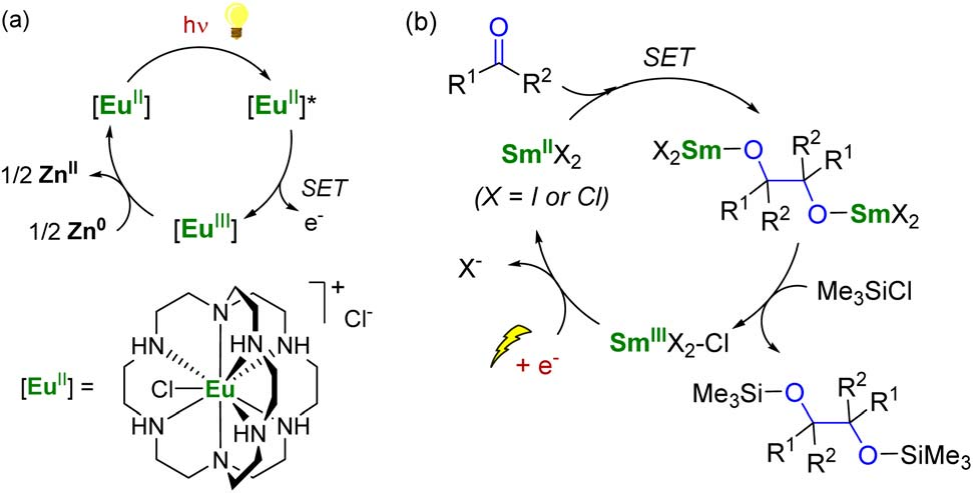
(a) (Photo)catalytic cycle with a Eu^II^ complex (a)^113^ and electrochemical approach for the recycling of Sm^II^ species (b).^133^

In the field of divalent lanthanides, only very scarce examples have been reported, mainly focusing on the recycling of SmI_2_ when used in catalytic amounts.^124^ Three different strategies have been described to reduce the Sm^III^ species back into Sm^II^. The most common strategy corresponds to a chemical approach involving the addition of a sacrificial reductant. For example, elemental magnesium,^125–129^ mischmetal (a low-cost alloy of the light lanthanides)^130,131^ or Zn/Hg amalgam^132^ have been used for this purpose (Scheme 3). Typically, a silyl-based electrophilic reagent (Me_3_SiCl, Me_3_SiOTf) is added to trap the anionic organic product and favor its decoordination from the oxophilic metal center. This strategy involving an external reductant has been successfully applied in the first catalytic visible-light-promoted reductive coupling of benzyl chloride by a Eu^II^ complex coordinated by an azacryptand macrocyclic ligand (Scheme 4).^113^ In this reaction, Zn^0^ powder is used as a sacrificial reducing agent to reduce the Eu^III^ complex formed after the SET step into its divalent Eu^II^ analog.

Although the chemical reduction approach is powerful, developing alternative methods that avoid using terminal reductants in stoichiometric quantities would be desirable. In this context, the group of Mellah has investigated electrochemical methods to reduce Sm^III^ species back into Sm^II^ at the surface of an electrode (Scheme 4). The corresponding system has been applied in carbon–carbon coupling reactions such as pinacol formation and Barbier-type reactions,^133^ in the reduction of nitrobenzenes into azobenzenes,^134^ in the carboxylation of benzyl halides with CO_2_,^135^ and reductive alkoxylation of phthalimides into isoindolinone derivatives (using 10–20 mol% of Sm^II^ species).^136^ The best results were obtained using a Sm metal electrode, which, acting as a cathode, is not consumed during electrolysis. Other more conventional cathode materials such as platinum, carbon, nickel, lead, or stainless steel did not lead to the successful regeneration of the Sm^II^ active species. The extension of the electrochemical strategy to the recycling of divalent organometallic complexes, such as those depicted in Fig. 4, may be very promising for developing new electrocatalytic transformations with organolanthanide complexes.

**Scheme 5:**
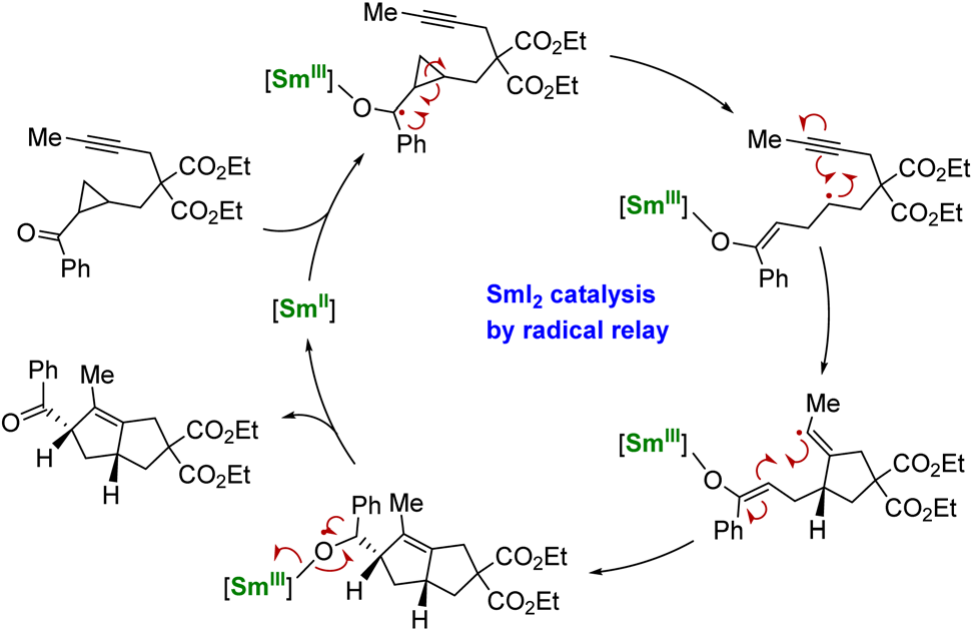
Schematic catalytic cycle of the Sm^ii^-induced electron transfer followed by cyclization and back electron transfer.^137^

Using another strategy, the group of Procter demonstrated that a radical relay approach could allow the use of SmI_2_ in catalytic amounts for organic transformations.^137,138^ In this strategy, no external reducing agent is necessary as the Sm^II^ species is regenerated through back electron transfer from a negatively charged organic intermediate (Scheme 5). However, this method requires the particular design of phenone substrates compatible with radical relay cyclization cascades and is, therefore, currently limited to very specific molecules. Despite its elegance, this strategy shows a heavy dependence on the nature of the substrate and the relative stability of the ketyl radical intermediates: tuning the ligand may be a key to developing this peculiar chemistry.^138^

Indeed, similar back electron transfer reactions between a radical anion intermediate and a Sm^III^ metal center have been observed in several instances in organosamarium complexes but highly depend on the reaction conditions and nature of the substrates. For example, the reduction of diphenylacetylene,^139^ conjugated alkenes,^140,141^ and polycyclic aromatic hydrocarbons^142^ by Sm(Cp*)_2_(thf) was found to be reversible depending on the solvent used. While the different substrates are reduced in hydrocarbon solvents, leading to the formation of dinuclear Sm^III^ complexes, these reactions can be reversed by adding THF, resulting in the regeneration of samarocene and the corresponding free alkyne/alkene. Similar observations involving Yb^II^ or Sm^II^ organometallic complexes have also been reported using different redox-active ligands.^51,143,144^

**Scheme 6:**
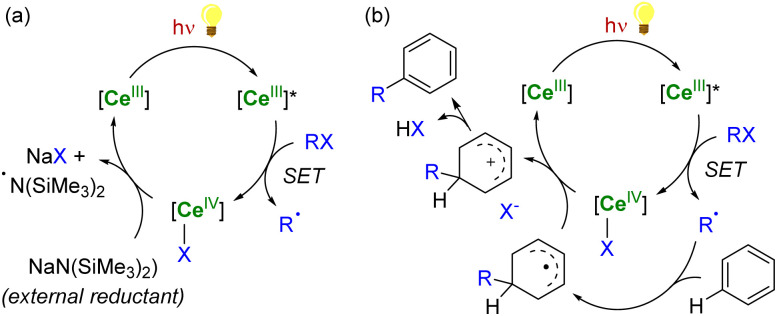
Schematic (photo)catalytic cycle involving the Ce(iii/iv) couple with (a) or without (b) the use of an external sacrificial reductant.^91,94^

In the case of Ce^IV^ species obtained upon SET reductions induced by photoexcited Ce^III^ complexes, the regeneration of the latter can also be achieved by different strategies. External reductants have been used to recycle the photoactive Ce^III^ species, which allowed the use of a catalytic amount of cerium photosensitizer. For example, MN(SiMe_3_)_2_ (M = Na, K) has been used as a sacrificial reductant that effectively reduces the Ce^IV^–Cl products to Ce^III^ products through the formation of an aminyl ˙N(SiMe_3_)_2_ radical (Scheme 6a).^91,94^ The presence of an external reductant, such as Ce or Zn metal powder, was sometimes necessary to quench the aminyl radical and prevent the formation of by-products.^91^ Depending on the nature of the substrate, the organic radical generated after the SET reduction step may also be oxidized by the Ce^IV^ complex, which provides a way to regenerate the photoactive Ce^III^ complexes for applications in catalytic transformations (Scheme 6b).^94^

**Scheme 7:**
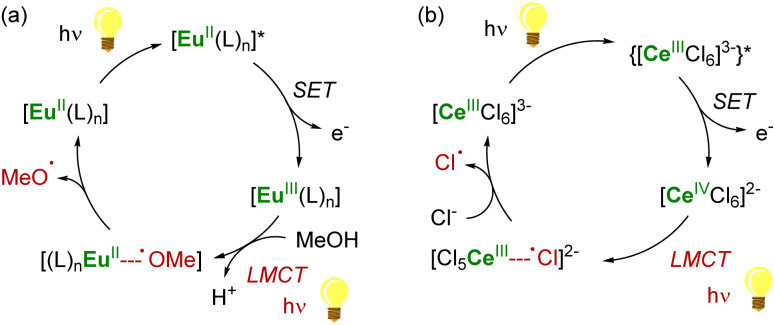
LMCT-induced homolysis in Eu^III^/Eu^II^ (a)^146,149^ and Ce^IV^/Ce^III^ (photo)catalysis (b).^90^

Another strategy for regenerating Ln^III^ photosensitizers, which has been especially developed over the last decade, corresponds to the photoreduction of the corresponding Ln^IV^ complexes with the concomitant formation of organic radicals. This strategy may be used for lanthanide compounds featuring a relatively stable +IV oxidation state, such as Ce^IV^ and Tb^IV^ species, with 4f^0^ and 4f^7^ electron configurations, respectively. These oxidizing ions display low-energy metal-to-ligand charge transfer (LMCT) absorptions, typically in the near UV and visible region. The corresponding excited states are usually not emissive but reactive.^87^ Upon irradiation, Ce^IV^ complexes undergo LMCT transitions which promote homolysis of the metal–ligand bond, leading to the photoreduction of the lanthanide center together with the generation of reactive ligand-centered radicals (Scheme 7). It should also be mentioned that this photochemical strategy had already been reported more than 30 years ago for recycling Ln^II^ (Ln = Sm, Eu, Yb) species for catalytic applications,^145–147^ but has only recently seen a renaissance in Ce chemistry.^90,148,149^

Using this procedure, reactive heteroatom-centered radical species such as carboxyl,^150^ alkoxy^151^ and chlorine^92,152^ radicals can be formed and used to activate other substrates under mild conditions. This strategy has recently been employed by Zuo and Schelter in several photocatalytic transformations induced by soluble cerium complexes and relying on the +IV/+III redox couple of the cerium center.^90,148,149^ For example, cerium photoredox catalysis has been applied in photocatalytic dehydrogenation of amines, C–C bond cleavage and functionalization of alcohols,^153–157^ C–H activation of alkanes^152,158,159^ and functionalization of aryl substrates.^93^ Although commercially available Ce^III^ salts (chlorides or triflates) are typically used in these photocatalytic reactions, the development of new stable Ln^IV^ complexes may be attractive for photocatalytic applications and to control the homolysis of the Ln^IV^–ligand bonds.

**Fig. 5:**
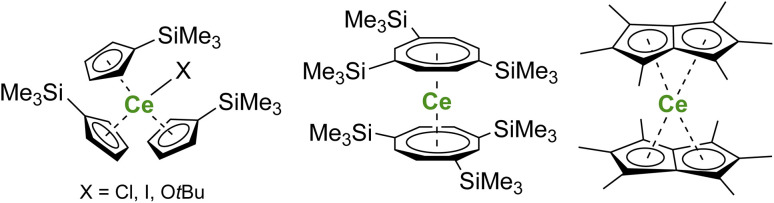
Examples of Ce^IV^ metallocene complexes.

In organometallic chemistry, synthesizing organocerium(iv) complexes is challenging, which can be traced back to the strong oxidizing character of the Ce^IV^ ion and the reducing properties of carbon-based anionic ligands.^160^ Therefore, only limited examples of organometallic cerium(iv) complexes have been reported in the literature, and the development of an organometallic scaffold allowing the stabilization of Ce^IV^ species is highly desirable. To date, organometallic Ce^IV^ complexes have been mostly limited to metallocene structures with examples of Ce^IV^ complexes bearing monoanionic cyclopentadienyl (Cp) ligands,^161–167^ dianionic cyclooctatetraenyl rings,^30,31,168,169^ and substituted pentalene dianionic ligands^170,171^ (Fig. 5). Improvement of the kinetic stability of Ce^IV^ organometallic complexes, especially by tuning the steric properties of the ligands, may be a direction of future work to go toward catalysis by organocerium complexes.

### Single-ion magnets and quantum technologies

#### Single-molecule magnets (SMMs) and f-elements

A single-molecule magnet is a molecule that can behave as a magnet below a given temperature, allowing a coercive field, which is a typical property of bulk magnetic materials. The first representative of this family was discovered in 1993 and corresponded to a molecular manganese cluster.^172^ Initially, the chosen strategy was to increase the spin state of the molecule, playing with strong metal–metal magnetic interactions to increase the barrier rapidly. Those studies showed that the overall barrier to spin reversal was far from the only important criterion.^173^ For this reason, lanthanide ions which had been ignored mainly because of their poor metal–metal magnetic communication, were placed back under the spotlight. Indeed, 4f-elements have strong magnetization thanks to the unquenched orbital moment. Due to the spin–orbit coupling, the good quantum number is *J*, and m_*J*_ values can reach ±15/2 with a strong anisotropy under a magnetic field.^174^

**Fig. 6:**
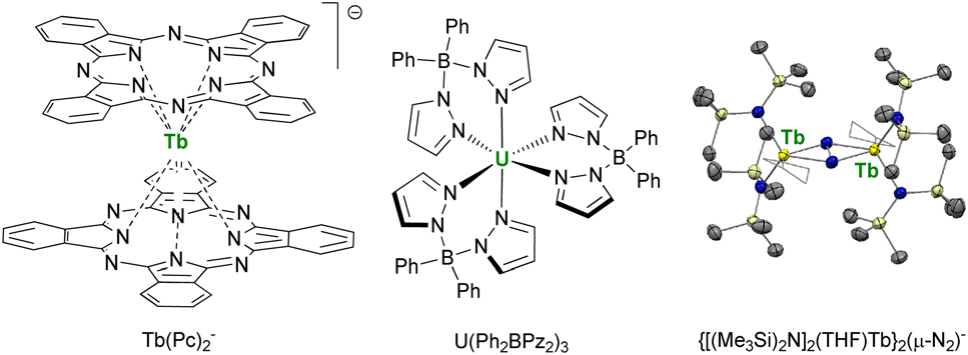
Molecular structures of Tb(Pc)_2_^−^,^70^U(Ph_2_BPz_2_)_3_,^176,177^ and ORTEP plot of {[(Me_3_Si)_2_N]_2_(THF)Tb}_2_(μ-N_2_)^−^,^180^ (terbium atoms are in yellow, nitrogen atoms in blue, silicon atoms in pale yellow, carbon atoms in grey, hydrogen atoms are not represented, and coordinated thf molecules are represented in wireframe).

This point was demonstrated in 2003 by Ishikawa *et al.* with a single-metal ion complex of Tb bis-phthalocyanine Tb(Pc)_2_^−^ (Fig. 6),^70,175^ which presented a remanent field under zero applied magnetic field. This critical study also underlined that the relaxation paths were multiple and that the surrounding of the central metal ion was crucial for maximizing the anisotropy and preventing the under-barrier relaxation sources from excited m_*J*_ states. The single-ion-magnet property was extended to 5f-ions with the neutral diphenyl bis(pyrazolyl borate) complex U(Ph_2_BPz_2_)_3_.^176,177^ However, a key challenge remained: to maximize the metal–metal magnetic exchange in f-elements combining anisotropy and high ground m_*J*_ value. This strategy particularly advanced by using bridging radical ligands, such as N_2_^3−^ (Fig. 6), for which the strong magnetic exchange with f-elements provides strong metal–metal alignment and, inevitably, single-magnet properties.^178–180^ This powerful strategy is currently still used for the design of f-element single-molecule magnets, particularly with organometallic fragments (*vide infra*).^181^

These early studies strongly emphasized two points: the control of the ground m_*J*_ value is critical for maximizing the anisotropy. In contrast, the nature and energy of the excited m_*J*_ states are critical for controlling the relaxation paths. From these starting points, Rinehart and Long elaborated a comprehensive description of the relationship between the coordination environment of the 4f-elements and the relative energies of the m_*J*_ states using a simple electrostatic model.^71^ This article was followed by a series of similar important notions,^182^ which moved the field to the rational design of SMMs. From then on, the unique structural arrangements accessible using organometallic complexes started to play a more significant role.

#### Single-ion magnet design

The prediction model shows three categories of lanthanide ions: the isotropic ions (Gd^III^, Eu^II^), the oblate-shaped ions, and the prolate ions.^71^ The first category is not interesting for the design of SMMs; the second requires a localized axial field to favor an isolated maximal m_*J*_ state value while equatorial fields are most desirable for the third category. More precisely, each ion fits best with one specific structural arrangement. If one focuses on the largest m_J_ ground state, the dysprosium(iii) ion requires a strongly localized axial field. In contrast, the erbium(iii) ion requires typical sandwich π-coordination, in which the overall charge surrounds the metal ion.^182^

**Fig. 7:**
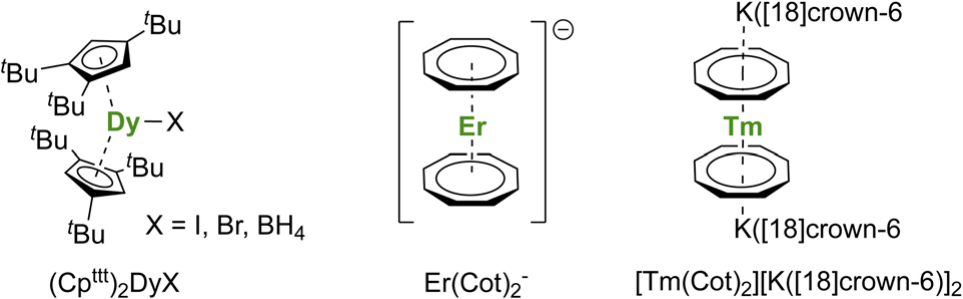
Molecular structures of (Cp^ttt^)_2_Dy(μ-I)K([18]crown-6),^60^Er(Cot)_2_^−^,^183^ and [Tm(Cot)_2_][K([18]crown-6)]_2_.^76^

A short review of the compounds known from the organolanthanide chemistry that would have an approaching adapted structure established that the (Cp^ttt^)_2_Dy(X) (X = Br, I, BH_4_) complexes made in 2007 by Nief and co-workers (Fig. 7),^60^ and the Er^iii^ representative of the K[Ln(Cot)_2_] family, pioneered by Streitwieser,^29^ would meet most criteria. In the latter, the chelation of the potassium ion led to the isolated Er(Cot)_2_^−^ anion (Fig. 7) that was reported with record SMMs properties at the time.^183^ The formation of heteroleptic neutral π-sandwich complexes was also investigated and led to good SMMs properties,^184,185^ particularly with the large monoanionic Cnt ligand.^186,187^ The Cot–Er pair is highly relevant as an excellent example of the perfect adequation between the metal and the ligand coordination for maximal anisotropy: this is referred to as ligand–metal pair anisotropy.^188,189^ The concept has been extended to divalent thulium, an ion with prolate-shaped density. However, the SMM properties of the corresponding complex (Fig. 7) remain very modest due to the decrease of the ground m_*J*_ states to ±7/2.^76^

**Fig. 8:**
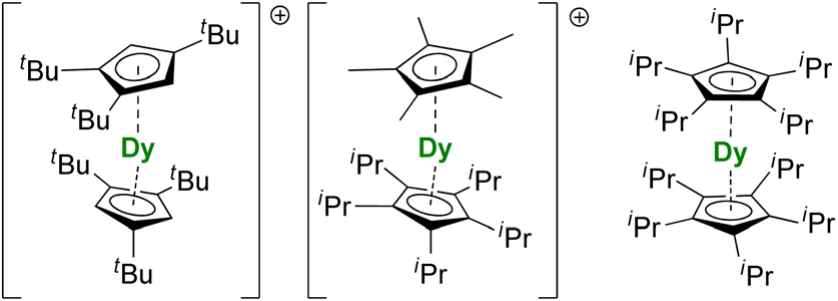
Molecular structures [Dy(Cp^ttt^)_2_]^+^,^72,73^[Dy(Cp*)(Cp^iPr5^)]^+^,^74^ and Dy(Cp^iPr5^)_2_.^193^

For the Dy(iii) case, building on the previous observations that steric bulk on the Cp substituents could provide linearity to the corresponding sandwich complexes,^77,120,190^ the Cp^ttt^ was found a suitable ligand that enforces axial ligand field. However, a problem remained owing to the presence of the halide counteranion coordinated in the equatorial sphere, even upon reduction.^60^ The key solution consisted of using [H(SiEt_3_)_2_]^+^[B(C_6_F_5_)_4_]^−^ as a halide abstraction reagent, leading to the separated [Dy(Cp^ttt^)_2_]^+^[B(C_6_F_5_)_4_]^−^ ion-pair (Fig. 8).^72,73^ The SMM properties of this molecule hammered the previous record with a 60 K blocking temperature and a 1541(11) cm^−1^ energy barrier. A few months later, this landmark was increased to 80 K, *i.e.* above the liquid nitrogen temperature, by increasing the bulk of the ligand with the use of the Cp^iPr5^ ligand (Fig. 8),^74^ a ligand previously used for the formation of Ln(Cp^iPr5^)_2_ (Ln = Sm, Eu, Yb).^77,191,192^ The modulation of the Cp substituents leads to variations in the complexes' geometry, directly impacting the magnetic properties. Additionally, the strategy was extended to non-classical divalent lanthanide complexes featuring a linear geometry (Fig. 8), which is remarkable, considering the larger size of the Ln^ii^ ions.^193^ However, reducing the lanthanide ions does not necessarily fill the f-shell, resulting in complications in the magnetic analysis because of 5d and 6s contributions.

In addition, a few lanthanide complexes bearing aromatic rings with sizes other than *C*_*5*_ or *C*_*8*_ also showed interesting SMM properties.^194^ One intriguing question remains in the case of the Ln(Bz^ttt^)_2_ family of complexes (Fig. 3) reported by Cloke and co-workers, since the ligand is intermediate between a large ring and a strongly localized aromatic anion.^79–82^ In these compounds, the electron count and charge on the ligand will depend on the actual oxidation state of the lanthanide ion.

### Radical bridge for strong metal–metal enhancement

**Fig. 9:**
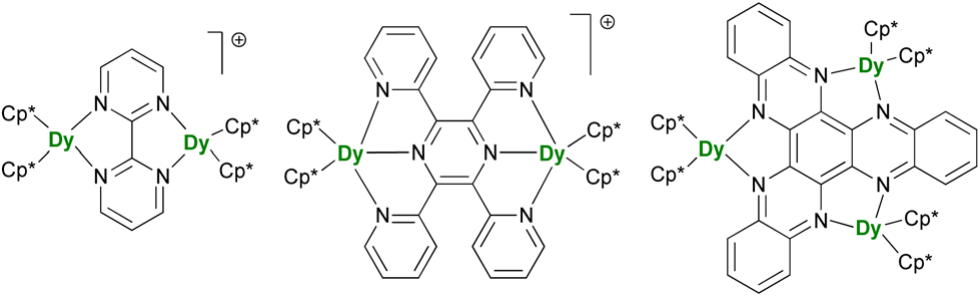
Molecular structures of [{(Cp*)_2_Dy}_2_(μ-bipym)]^+^,^196^[{(Cp*)_2_Dy}_2_(μ-tppz)]^+^,^197^ and (Cp*)_6_Dy_3_(μ_3_-HAN).^198^

After the description of radical-induced metal alignment with reduced dinitrogen radicals, a series of compounds was reported with the objective of increasing the energetic barrier. For such a purpose, the target of choice corresponds to soluble and versatile lanthanide fragments associated with redox-active bridging ligands. For example, the N_2_^3−^ bridging ligand can be easily replaced by another π-accepting ligand such as N-aromatic heterocycles. The known {(Cp*)_2_Ln}_2_(μ-bipym) (bipym = bipyrimidine) complex was an obvious choice because of the symmetrical and bridging nature of the ligand,^195^ which would compare well with the terminal bipyridine ligand.^46,47^ However, this complex, obtained upon reaction of two equivalents of divalent lanthanide precursor with free bipym, features a doubly reduced dianion bridge. Therefore, no radical is present on the ligand, which renders the corresponding complex of limited interest in terms of magnetic exchange. The more appealing radical bridged [{(Cp*)_2_Ln}_2_(μ-bipym)]^+^ cations (Fig. 9) could be obtained by first coordination of bipym to cationic {(Cp*)_2_Ln}^+^ fragments, followed by single-electron reduction. As a result, both lanthanide ions are trivalent, and the bipym is in a radical mono-anionic form.^196^ These compounds exhibit SMM behavior, with barriers essentially smaller than those in the N_2_^3−^-radical-bridged analogs,^179,180^ but can be compared to the values obtained in complexes with larger ligands such as the 2,3,5,6-tetra(2-pyridyl)pyrazine (tppz)^197^ or in related trimetallic arrangements using the μ_3_-hexaazatrinaphthylene (HAN)^198^ ligand (Fig. 9). The great advantage of such systems is that both the organometallic fragment and the bridging ligand can be easily and independently tuned: the influence of the coordination sphere around the lanthanide ion,^199^ as well as that of the bridging ligand electronic properties, through the use of electron-donating and withdrawing substituents, could be evaluated.^200^ The two conclusions of this remarkable series of works by Demir and Long are that the best SMMs should possess maximal magnetic exchange while retaining as much as possible an axial field. The ligand's size and electronics (nature of the bonding) are both critical in this matter.

An ideal way to embrace both conditions would be to have one single electron placed between two lanthanide ions, each possessing a strong and highly localized axial field. This strategy was recently addressed by Long and coworkers, who designed the {Cp^iPr5^LnI}_2_(μ-I)_2_ dimeric structures in which the Cp^iPr5^ ligands are each capping one lanthanide ion. The rest of the metal coordination sphere was completed by iodide ligands.^201^ The single electron that would favor the magnetic exchange was placed by simply reducing one metal center to afford a mixed-valent organometallic complex, {Cp^iPr5^Ln}_2_(μ-I)_3_, in which both metal ions can magnetically communicate. When the extra electron is placed in the f-shell, the coupling is not strong; however, when it is placed in the d-shell, the coupling is maximum, and the Hund's rules are verified. As a result, all metallic spins align, leading to a giant coercive field and ultrahard magnetism at high temperatures (60 K).^201^

Beyond these remarkable results, there are a few take-home points: the blocking temperature and effective barrier can reach very high values so that feasible quantum applications may be considered in the future. The ligand design is a crucial parameter to find adapted metal–ligand anisotropy, and it seems that π-coordinating ligands are particularly well-suited for designing high-performance SMMs and providing the correct geometry. These characteristics rely on how the relaxation paths are selected, an essential key for designing future quantum devices.

### Relaxation paths analysis

As mentioned above, the thermally activated barrier, corresponding to the Orbach model, is not satisfactory as a sole criterion for describing the SMM properties.^202^ This is mainly due to possible under-the-barrier magnetic relaxation paths *via* Raman (spin phonon) and quantum tunneling, which need to be unraveled. It is essential to rationalize how these processes can be avoided using an appropriate molecular design.^203^

Several approaches were proposed, such as using rigid ligands to increase the energy of the phonons and avoid them from meeting with the energies of the m_*J*_ excited states.^202,204^ A second approach, which is appealing, consists in preventing resonance phonon transitions from the magnetic energy window. For this purpose, it is necessary to relate the structural features to the energetic ladder, which has been attempted on several occasions and is an important current objective in the theoretical chemistry community.^205^ As such, in the dysprosocenium series, the reasons for recording SMM behavior can be understood by two distinct phenomena. First, the near linear arrangement of the ligands allows maximal splitting of the energy states. In their article, Chilton *et al.* argued that the larger crystal field splitting is the primary driver of the slower relaxation rate for [Dy(Cp*)(Cp^iPr5^)]^+^ compared to [Dy(Cp^ttt^)_2_]^+^.^205^ Then, the first complex also has the advantage of possessing electronic states that are off-resonance with vibronic states. The investigation of such an interplay between the structural features and the electronic structure is of great importance for bringing the rational design of SMM even further. Combining theory and luminescence spectroscopy could be instrumental in rationalizing a potential correlation.^206,207^

Additionally, the development of heterocyclic ligands such as phospholyl or new aminoborolide ligands could bring new data to enable broader comprehension of the magnetic relaxation pathways.^208–210^

### Quantum devices

A very promising application of the structure–physical property relationship made over the most recent years lies in the development of quantum devices. Molecular qubits are increasingly cited as potential tools for tailoring quantum algorithms^211^ since they are highly tunable and would be easily excited by external physical stimuli, such as microwaves or light.^212^ Organometallic compounds have recently been highlighted as potent molecules to pursue in this direction.^213^

**Fig. 10:**
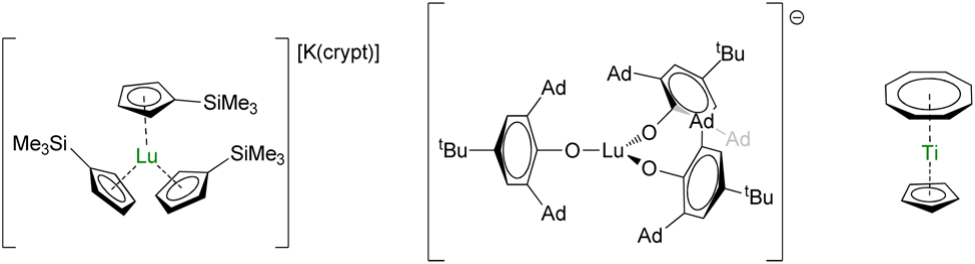
Molecular structures of [Lu(Cp′)_3_][K(crypt)],^217^Lu(OAr*)_3_^−^,^218^ and (Cp)Ti(Cot).^213^

Aromi and Sessoli proposed lanthanide coordination compounds in this direction,^214–216^ and the report of the electronic relaxation of Lu(Cp′)_3_^−^ (Fig. 10) further increased the interest.^217,218^ Indeed, the sizeable hyperfine coupling constant, *A*_ISO_ = 428.5 G, can be associated with a significant s-character to the magnetic orbital carrying the spin. In turn, spin–orbit coupling is minimized and spin–lattice relaxation is reduced in systems with *J* > 1/2. Maximizing *A*_ISO_ is this key, which can be done by modifying the ligand set around the metal center. With this in mind, high-field EPR measurements were performed on the bulky tris(aryloxide) Lu(OAr*)_3_^−^ (OAr* = 2,6-Ad_2_-4^*t*^Bu-C_6_H_2_O, Ad is for adamantyl, Fig. 10), which displayed a massive *A*_ISO_ value of 3467 MHz, similar to that in a Bi^ii^ radical,^219^ with giant clock transition.^218^ The control of the bonding is primordial for manipulating the electronic properties and systematic structure/properties correlations will be necessary for the design of future molecules or reinvestigating ghosts from the past.

Materials, such as Er^3+^:CaWO_4_ and Yb^3+^:YSO (YSO = yttrium orthosilicate, Y_2_SiO_5_),^220,221^ have shown interesting coherence properties, and the molecular extension to these molecules shall bring much information to the field. The use of carbon-based ligands, which possess 99% ^12^C (*I* = 0) and only 1% ^13^C (*I* = 1/2) and are relatively easy to deuterate at specific positions, should bring valuable information. In contrast, access to multimetallic compounds with close-but-different ligand fields would be appealing. These requirements are reminiscent of the lanthanide multiple-decker chemistry developed with large aromatic ligands such as Cot and its substituted analogs.^25,222^ Monometallic sandwich compounds have already shown promising results, as in the case of titanium for example, which also demonstrated that the presence of protons could not be as critical as anticipated with such ligands (Fig. 10).^213^ The use of multiple oxidation states, such as in divalent thulium, a simple f^13^ (*I* = 1/2) ion which ground m_*J*_ state and hyperfine coupling constants can be easily tuned,^223,224^ is also a future topic of interest while the population of the 5d-shell (often hybridized with the 6s-orbital) in non-classical divalent lanthanides will provide many future research directions.^76,225^

## Conclusions

What started as a search for peculiar geometry promptly developed into a vast array of original chemistry, from single electron reductants to polymerization catalysts. The interplay between the aromatic ligand and the lanthanide center was better understood through these different applications. This allowed, in turn, a rational tuning of the ligand to harness the intrinsic magnetic and electronic properties of the metallic center, leading to the recent breakthrough in these fields. Given the constant back-and-forth shuttle between old structures and new outlooks, highlighted in this perspective, a modern look at classic lanthanide organometallic compounds from the past will likely lead to a bright future.

## Author contributions

GN designed the structure of the perspective. All authors contributed to the writing of the article.

## Conflicts of interest

There are no conflicts to declare.

## Supplementary Material
